# Detecting DNA damage in stored blood samples

**DOI:** 10.1007/s12024-022-00549-3

**Published:** 2022-11-10

**Authors:** Kristina Schulze Johann, Hannah Bauer, Peter Wiegand, Heidi Pfeiffer, Marielle Vennemann

**Affiliations:** 1 Institute of Legal Medicine, University of Münster, Röntgenstr. 23, 48149 Münster, Germany; 2 Institute of Legal Medicine, University of Ulm, Albert-Einstein-Allee 23, 89081 Ulm, Germany

**Keywords:** Degradation, Quantification, UNG, Forensic DNA analysis

## Abstract

Several commercially available quantitative real-time PCR (qPCR) systems enable highly sensitive detection of human DNA and provide a degradation index (DI) to assess DNA quality. From routine casework in forensic genetics, it was observed that DNA degradation in forensic samples such as blood samples stored under sub-optimal conditions leads to visible effects in multiplex analyses of short tandem repeat markers (STRs) due to decreased amplification efficiencies in longer amplicons. It was further noticed that degradation indices often remain below the value that is considered to be critical. Thus, the aim of this work was to systematically analyze this effect and to compare conventional qPCR assays with a modified qPCR approach using uracil DNA glycosylase (UNG) and DNA quality assessment methods based on electrophoresis. Blood samples were stored at three different storage temperatures for up to 316 days. Significantly increased DNA recovery was observed from samples stored at high temperatures (37 °C) compared samples stored at room temperature and 4 °C. We observed typical effects of degradation in STR analyses but no correlation between DI and storage time in any of the storage conditions. Adding UNG slightly increased the sensitivity of detecting DNA degradation in one of the qPCR kits used in this study. This observation was not confirmed when using a second qPCR system. Electrophoretic systems did also not reveal significant correlations between integrity values and time. Methods for detecting DNA degradation are usually limited to the detection of DNA fragmentation, and we conclude that degradation affecting forensic STR typing is more complex.

## Introduction

**Fig. 1:**
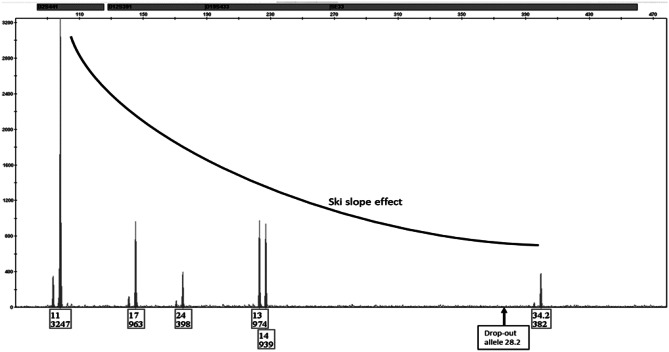
Ski-slope effect; DNA degradation leads to fragmentation of the DNA strand, which results in a lower amplification efficiency of longer amplicons compared to shorter amplicons with increased risk of drop-out. Labels show allele calls and relative fluorescence units (rfu)

Over the past decades, DNA profiling of biological crime scene traces by multiplex PCR analysis of short tandem repeat (STR) markers has become a very important tool in the investigation of crime. One of the main challenges forensic scientists encounter when working with biological trace evidence is DNA fragmentation resulting from degradation through chemical damage. Various external factors such as ultraviolet light, radiation, temperature, or humidity damage DNA structure due to the DNA’s limited chemical stability. One of the main chemical reactions by which DNA is damaged is hydrolytic degradation, the cleavage of chemical bonds by the addition of water [1–4].There are two main mechanisms by which hydrolysis attacks DNA integrity, that of base loss from the 2′-deoxyribose backbone and that of deamination. Hydrolysis of the DNA backbone attacks the linkage between the deoxyribose carbon atom and the base and is the main reason for DNA degradation in dead tissue [5]. When the DNA bases cytosine, adenine, and guanine undergo the process of spontaneous deamination, hence the removal of an amino group, they will be converted to uracil, hypoxanthine, and xanthine, respectively, and ammonia (this process is reviewed here: [6]). Deamination of DNA bases leads to sequence variation and structure instability: During PCR, uracil is known to pair with adenine, resulting in C-T transitions in the amplicon [7]. Hypoxanthine is known to have a pairing preference with cytosine, resulting in A-G transition [8]. The mechanism by which deamination of cytosine into uracil is repaired is by removal of uracil by uracil DNA glycosylase (UNG). This results in the generation of an abasic site, which are more prone to spontaneous and glycosylase-mediated DNA strand breaks [9]. Mechanisms described above may ultimately lead to DNA strand breaks. Fragmentation of the DNA strand can result in decreasing PCR efficiency in longer amplicons. In multiplex STR amplification, this phenomenon is well known as “ski-slope effect” visible in electropherorgrams with high peak heights of short amplicons and low peak heights of longer amplicons that might become so low that drop-outs of amplicons occur [10]. Figure 1 shows an example of such a STR profile.

The effect of differing amplification efficiencies in multiplex STR analysis challenges the interpretation of STR typing results, particularly in more complex DNA mixtures: Degradation might lead to a high number of alleles in short amplicon STR markers and low numbers of alleles in longer amplicon markers, making it difficult to estimate the number of contributors to a mixture [11, 12]. This is further complicated if one component of the DNA mixture is degraded, while others are intact. Several DNA quantification tools based on quantitative real-time PCR now also incorporate the detection of DNA fragmentation. For example, the PowerQuant^®^ System (Promega) contains primers and probes for an autosomal target and a “degradation target” with the autosomal target (84 bp) being much shorter compared to the degradation target (296 bp) [13]. The amplification efficiency differs between these two targets in degraded samples but not in intact samples. Similarly, the Investigator Quantiplex^®^ Pro kit (Qiagen) contains an autosomal target of 91 bp and a “degradation target” of 353 bp [14], and the Quantifiler^®^ Trio DNA Quantification Kit (Thermo Fisher Scientific) contains an autosomal target of 80 bp and a “degradation target” of 214 bp [14]. In degraded samples, the DNA concentration detected with the degradation targets is expected to be lower than with the autosomal target. Thus, the relative value of DNA concentrations detected with both probes (“degradation index”) allows an assessment of DNA degradation before forensic STR analysis is performed.

In casework, however, we regularly observe serious degradation patterns with ski-slope effects and drop-outs of longer amplicons even though the degradation index of quantitative real-time PCR kits remains unobtrusive. The aim of this work was to systematically investigate the potential of quantitative real-time PCR to actually detect DNA degradation and whether or not the use of UNG might increase the sensitivity of these systems to detect degradation. In 1993, Lindahl described for the first time that during DNA degradation, hydrolysis may lead to deamination of cytosine creating uracil [5], making these positions potential targets for UNG.

Furthermore, methods using electrophoresis for assessing DNA quality were investigated as a potential alternative for detecting forensically relevant DNA degradation. Methods for DNA quality assessments based on electrophoresis are regularly used in massively parallel sequencing experiments to assess DNA fragment size distribution as a measure of degradation [15, 16]. Automated capillary electrophoresis and pulse-field capillary electrophoresis systems were used to detect DNA Integrity numbers DIN [17] and Genomic Quality Numbers (GQN) [18], respectively.

## Material and methods

### Sample collection and DNA extraction

Blood samples were collected from one individual only to eliminate the risk of interindividual variations caused, i.e., by the general health status of the donors. A blood sample was collected by venipuncture, using EDTA as an anti-coagulant. Spots of 40 µL of blood were immediately placed on sterile cellulose pads, gently inverting the blood tube several times every 4 spots. Pads were stored at three different conditions; room temperature (RT, ~ 21 °C, indoors, protected from direct sunlight), 4 °C (fridge), and 37 °C (incubator). Further information on sampling times and conditions are described below. As a positive control DNA was extracted and quantified immediately after collection. DNA was extracted using the Maxwell^®^ RSC Blood DNA Kit with the Maxwell^®^ 16 Forensic Instrument (Promega, Mannheim, Germany) and eluted in a final volume of 50 µL per blood spot. Extracts were stored at − 20 °C until further analysis.

### DNA degradation in multiplex STR analysis

To confirm that DNA degradation producing ski-slope effects is visible with the storage conditions chosen, samples from three time points (0 days, 21 days, and 83 days) were analyzed using the PowerPlex^®^ ESX 17 System (Promega) following manufacturer’s recommendations but using a total reaction volume of 12.5 µL. Amplicons were visualized using the 3500 Genetic Analyzer, and data were interpreted using the GeneMapper™ ID-X Software v 1.6 (Thermo Fisher Scientific, Darmstadt, Germany). Degradation indices (DI) were analyzed using the PowerQuant System (Promega, Madison, US) with 2 μL sample, 5 μL 2 × Master Mix, 0.5 μL 20 × Primer/Probe/IPC Mix, and HPLC-grade water to a reaction volume of 11 μL. qPCR was performed using the 7500 real-time PCR system with the HID Real-Time PCR Analysis Software v1.2 (Thermo Fisher Scientific).

### DNA degradation detection by forensic quantitative real-time PCR

For this part of the experiment, samples were stored for up to 83 days at three temperatures, and DNA was extracted every two or 3 days. Thus, 35 time points per temperature condition totaling 105 samples were analyzed. Per condition and time point, duplicate extractions using four spots of 40 µL blood each were extracted separately, and the eluates were subsequently pooled to a total volume of 200 µL per replicate. Samples were quantified using the PowerQuant^®^ System as described above. DNA concentration in each sample and respective degradation indices were used for data analysis.

### Influence of uracil DNA glycosylase on detection sensitivity

To investigate if the use of uracil DNA glycosylase enhances the sensitivity of detecting DNA degradation, blood spots on cellulose pads were stored at RT for up to 316 days and extracted at 82 time points during this period. DNA was extracted in duplicates at each time point: One DNA extract was treated with AmpErase™ Uracil N-Glycosylase (UNG, Thermo Fisher Scientific) for 20 min at 50 °C, while the other remained untreated. DNA concentration in each extract and respective degradation indices were analyzed using the PowerQuant System as described above.

To evaluate if results can be confirmed when using an alternative quantification kit, the UNG experiment was repeated using a subset of samples (stored at RT for up to 176 days with 53 time points). Both UNG-treated and untreated DNA extracts were quantified using the Investigator Quantiplex^®^ Pro Kit (Qiagen, Hilden, Germany) following manufacturer’s recommendations.

### Detection of DNA degradation by automated (pulse-field) electrophoresis

To investigate if such electrophoretic methods are better suited for detecting DNA degradation in forensic casework samples, the 4150 TapeStation system (Verogen, San Diego, USA) with the Genomic DNA ScreenTape assay and TapeStation analysis software was used to calculate DNA integrity numbers (DIN). Furthermore, the FEMTO Pulse automated pulsed-field CE instrument (Agilent, Waldbronn) was used with the 165 kb analysis kit and ProSize data analysis software (with a size threshold of 50 000 bp).

Blood spots were stored at three different temperatures (4 °C, 37 °C, and RT) for up to 26 days (TapeStation) or 37 days (Femto). TapeStation analysis was performed after 11 different time points and Femto analysis after 16 different time points, each in duplicates. TapeStation analyses were performed at Agilent Technologies and Femto Pulse analyses were performed at Genomics & Transcriptomics Laboratory (GTL) at Heinrich-Heine-University Düsseldorf.

### Statistical analysis

For all calculations and figures, mean values of replicates were used. Statistically significant differences between degradation indices of different storage temperatures or duration were calculated using the Mann–Whitney *U* test using IBM SPSS Statistics version 28.0.1.0. The Mann–Whitney *U* test was chosen because there are no strict requirements to data distribution like normal or bivariate distribution and it can be used even in small sample sizes. Correlation between degradation indices and time were calculated using Pearson correlation using Microsoft Excel 2016. A value of *p* < 0.005 was considered statistically significant.

## Results

### DNA degradation in multiplex STR analysis

Apart from the control sample (0 days), DNA degradation was observed in samples from all storage conditions after 21 and 83 days. As an example, Fig. 1 shows the results of the PowerPlex^®^ ESX amplification in a sample stored at 37 °C for 21 days. A ski-slope effect is visible, and a drop-out occurred of allele 28.2 in the longest amplicon representing SE33. The degradation indices (DI) of all samples remained below the critical value of 2 recommended by the manufacturer [13, 14]. The sample depicted in Fig. 1, for example, revealed a DI of 1.2.

### DNA degradation detection by forensic quantitative real-time PCR

**Fig. 2:**
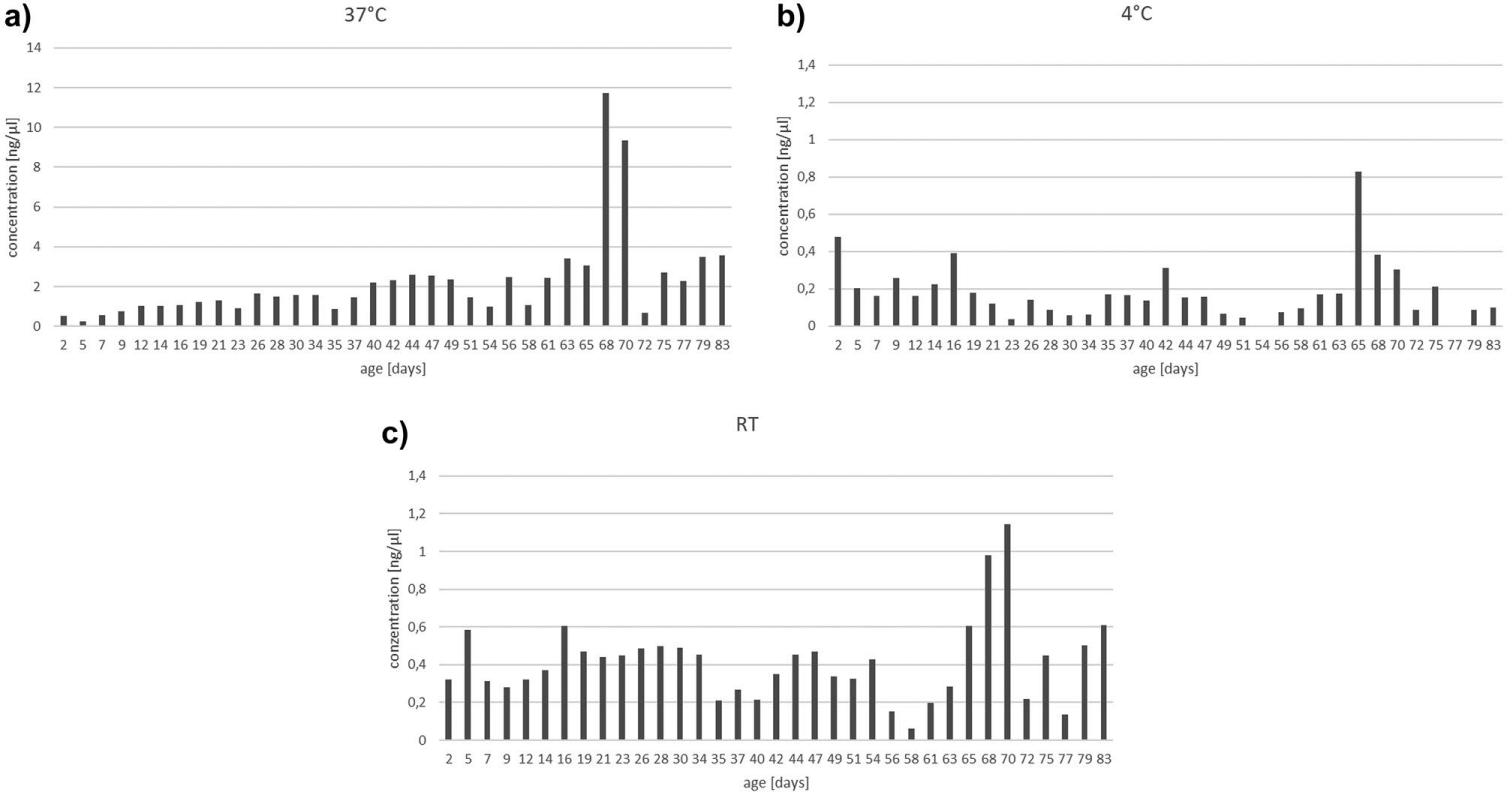
DNA concentration in ng/µL extracted from samples stored at three different temperatures (**a** 37 °C, **b** 4 °C, and **c** room temperature, RT). All samples were eluted in identical extraction volumes. Please note that the scaling of the *y*-axis in (**a**) differs from (**b**) and (**c**). No statistically significant loss of DNA over time was observed, but overall DNA concentrations were significantly higher in extracts from samples stored at 37 °C (**a**) compared to 4 °C (**b**) and RT (**c**)

First, total DNA concentration within each extract was quantified to detect any overall loss of DNA or extraction efficiency over time. No statistically significant DNA loss over time was observed for any of the three storage conditions (Fig. 2).

Statistically significant differences in DNA concentrations were observed between storage conditions with much higher DNA amounts extracted from samples stored at 37 °C compared to samples stored at 4 °C (*p* < 0.001) and RT (*p* < 0.001). Furthermore, instead of DNA loss over time, a clear increase of DNA concentrations over time was observed for samples stored at 37 °C (Fig. 2a, Pearson correlation coefficient 0.536, *p* = 0.0009). No such correlations were observed for samples stored at 4 °C and RT. Thus, DNA extraction efficiency was increased in samples stored at 37 °C for a prolonged time period.

**Fig. 3:**
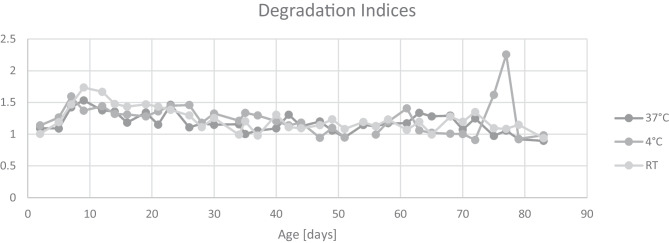
Degradation indices obtained by PowerQuant analysis over time under three different storage conditions

The DI was subsequently analyzed. At all storage conditions, an increase of DI was observed within the first seven days of storage, which seems to level out over time (Fig. 3). Over the total storage time, a slightly negative correlation between DI and time was observed for samples stored at 37 °C (Pearson correlation *r* =  − 0.464; *p* = 0.0049) and RT (*r* =  − 0.533; *p* = 0.0009). There is no significant correlation between DI and time in samples stored at 4 °C (*r* =  − 0.236; *p* = 0.172). This data confirms previous studies, for example one by Bulla et al., who reported loss of DNA quantity but not integrity in long-term storage of blood samples [19].

### Influence of uracil DNA glycosylase on detection sensitivity

**Fig. 4:**
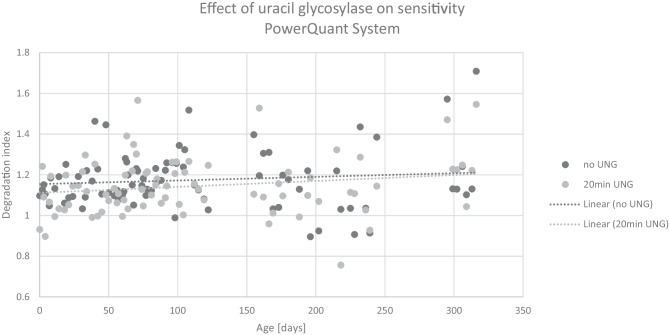
Effect of uracil DNA glycosylase on the detection sensitivity of DNA degradation with the PowerQuant System. No correlation was found between DI and storage time

Figure 4 shows the differences in DI between samples treated with UNG and untreated samples measured with the PowerQuant^®^ System. No clear trend of a significant increase in degradation index over time in samples treated with UNG was observed in either treated or untreated DNA extracts.

**Fig. 5:**
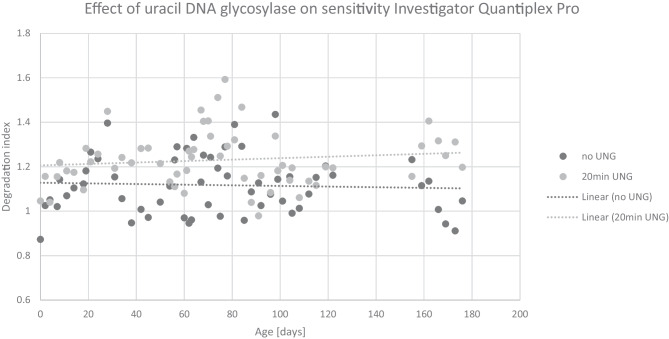
Effect of uracil DNA glycosylase on the detection sensitivity of DNA degradation with the Investigator Quantiplex Pro. No correlation was found between DI and storage time. But a generally higher DI was observed in samples treated with UNG compared to untreated samples (*p* < 0.001)

Results obtained with the Investigator Quantiplex Pro Kit (Fig. 5) provided a similar outcome to results obtained with PowerQuant System, and no statistically significant correlation between DI and storage time was found.

**Fig. 6:**
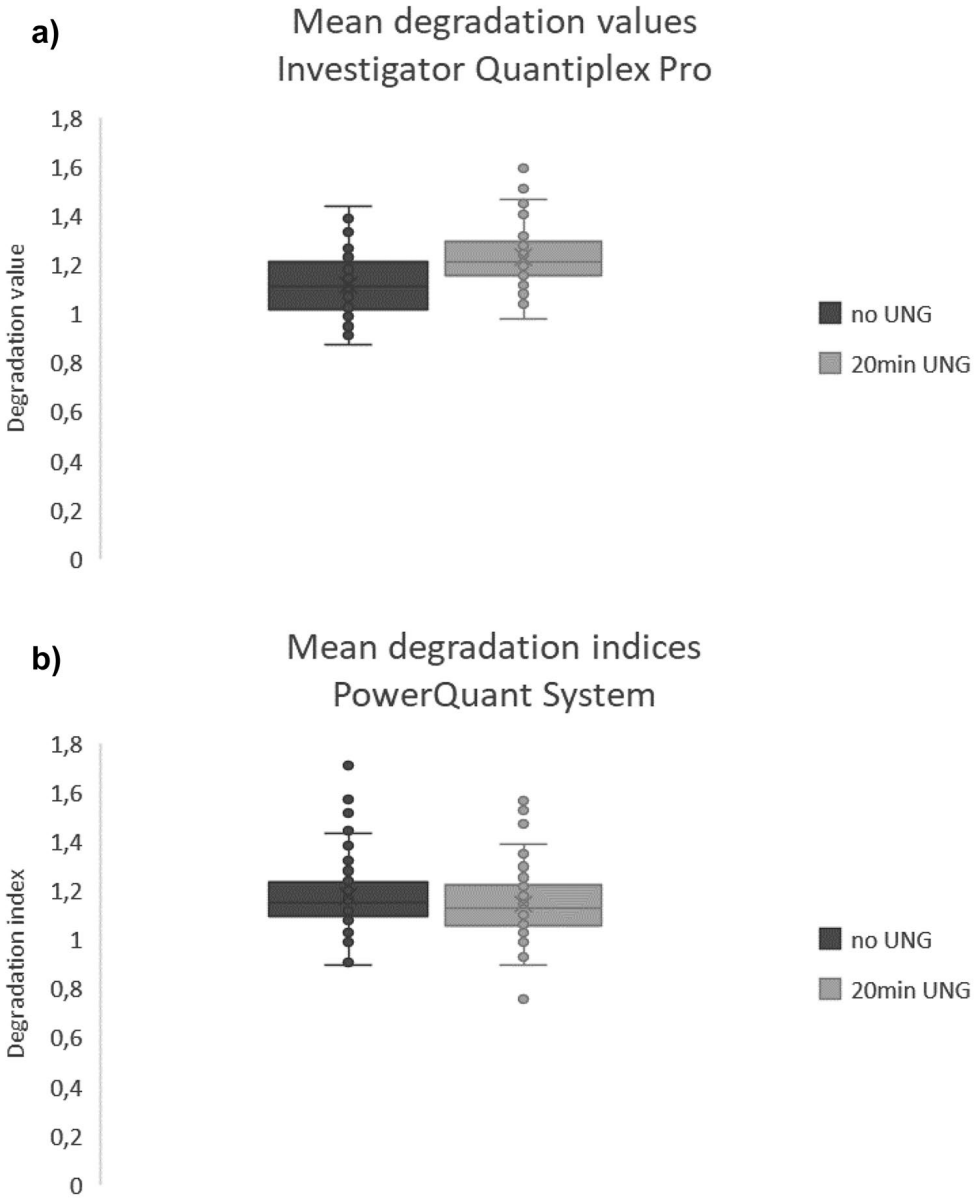
Overall mean degradation in samples treated with UNG compared to untreated, measured with the Investigator Quantiplex Pro (**a**) and PowerQuant System (**b**)

Samples analyzed with the Quantiplex^®^ Pro, however, showed a general difference in DI between samples treated with UNG and untreated samples (*p* < 0.001) indicating an increased sensitivity of degradation detection (Fig. 6a). This effect was not observed with the PowerQuant^®^ System (Fig. 6b).

### DNA degradation analysis by automated (pulse-field) electrophoresis

**Fig. 7:**
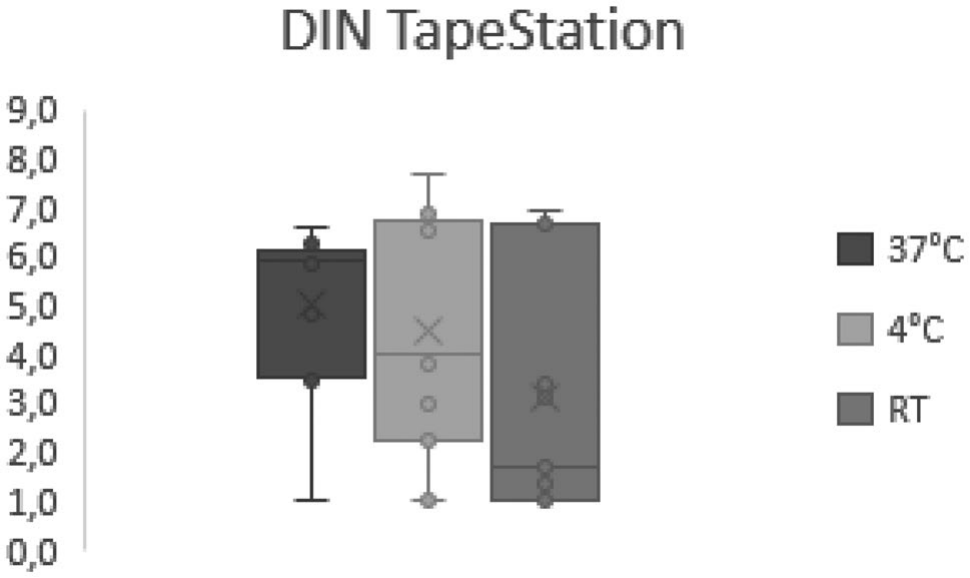
Boxplots showing DIN obtained with the TapeStation analysis in samples stored at different temperatures. A higher median DIN was observed in samples stored at 37 °C compared to 4 °C and RT, without reaching statistical significance

No significant correlation between DIN and storage time was observed at any of the temperatures (37 °C, *p* = 0.99; 4 °C, *p* = 0.2; RT, *p* = 0.85). Comparing DIN values between storage conditions, the median is considerably higher in samples stored at 37 °C compared to the other two storage conditions (Fig. 7).

**Fig. 8:**
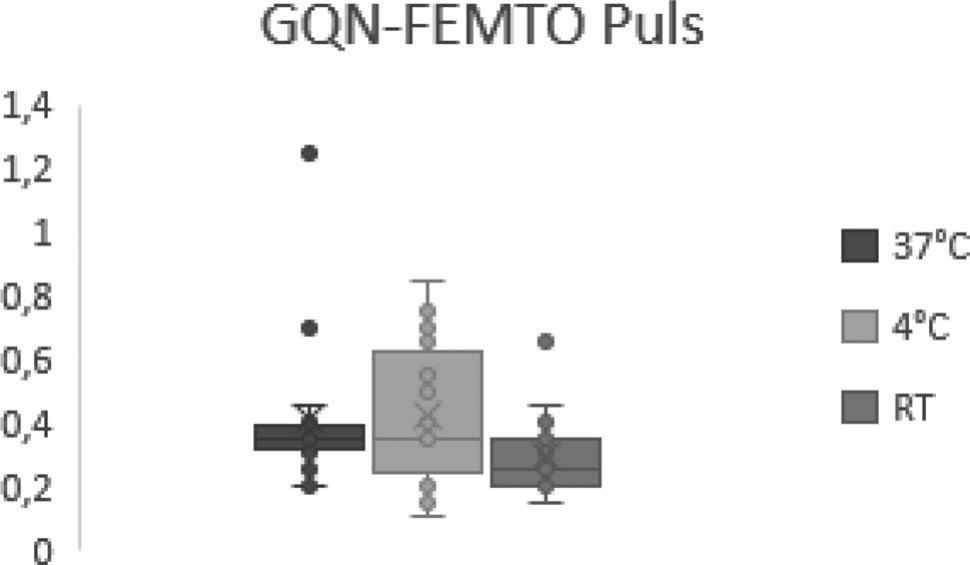
Boxplots showing GQN obtained with the Femto Pulse analysis in samples stored at different temperatures. No statistical significant differences between storage temperatures were observed

Similar to the DIN results, GQN does not correlate with the storage duration in any storage temperature (4 °C *p* = 0.3; 37 °C *p* = 0.98; RT *p* = 0.91). The mean GQN of the different storage temperatures does not show any significant differences between storage conditions (Fig. 8).

## Discussion

### DNA degradation in multiplex STR analysis

Our results confirm experiences obtained from real casework samples, in which degradation was observed in STR analyses, while DIs obtained from quantitative real-time PCR remained unobtrusive. Recently, Lin et al. observed that after artificially degrading DNA to fragment sizes of 300 to 500 bp, full or nearly full STR profiles were obtained. Even when DNA was extremely degraded to fragment sizes of 150 bp, still partial profiles were obtained. Degrading DNA at such a low level, autosomal, and degradation quantification values of all quantification kits compared in their study dropped and DI increased [20]. One explanation for the differing observations compared to our study might be that Lin et al. used artificial degradation until fragmentation was actually visible. Our study, on the other hand, did not artificially enhance degradation but relied on natural degradation over time. Thus, degradation might have been less severe in our samples and degradation mechanisms other than fragmentation might have occurred (see below).

### DNA degradation detection by forensic quantitative real-time PCR

No significant loss of DNA over time was observed in any of the storage conditions. On the contrary, DNA extraction efficiency was increased in samples stored at 37 °C for a prolonged time period. There is certainly no straight-forward explanation for this observation. We hypothesize, however, that drying of samples (loss of humidity) might play a crucial role here: Colder storage environments might lead to higher humidity, which, in turn, enables bacterial growth. Another hypothesis is that substances inhibiting extraction and/or detection processes might decompose faster at this temperature compared to DNA.

Several previous studies investigated the influence of storage temperature on DNA quantity and integrity in blood samples: For example, Al Rokayan found in 2000 that DNA extracted from blood samples showed higher molecular weight and less shearing if blood samples were stored at − 20 °C compared to samples stored at 4 °C and RT [21]. Huang et al. [22] described in 2017 a loss in DNA concentration from blood samples stored at 24 °C over 15 days and that this loss correlated with a decrease in white blood cell (WBC) counts [22]. They also reported that samples stored at a low temperature (4 °C) showed stronger loss in WBC counts compared to storage at 24 °C, explaining this by cell lysis due to stress [22]. Most laboratories use 4 °C for short-term storage of blood samples. Our results along with previously published data suggest that even for shorter storage periods of one to 2 weeks, storage at − 20 °C or an increased temperature is preferable over 4 °C.

No significant increase of DI over time was observed even though these samples showed signs of degradation in multiplex STR PCR analyses. Our data confirm previous studies, for example, by Bulla et al., who reported loss of DNA quantity but not integrity in long-term storage of blood samples [19]. Data obtained by Investigator Quantiplex Pro without UNG treatment also showed no increase in DI over time. This means that the limited sensitivity in detecting DNA degradation by qPCR is not kit specific but might be explained by a more general underlying principle, which we will discuss below.

Thus, DNA degradation affecting STR analysis proved to be surprisingly difficult to detect at all. This might be because we initiated natural degradation by storage over time, while previous studies mainly used artificially enhanced degradation. DI only detects DNA fragmentation, but DNA fragmentation might not be the only mechanism behind the ski-slope effect. Other mechanisms of DNA degradation might play a crucial role here. For example, chemical alterations of bases as described above might reduce the efficiency of primer binding due to mismatched positions in the presence of uracil and hypoxanthine instead of cytosine and adenine. Mismatched positions in primer binding sites are known to have a stronger effect on STR markers with longer amplicons, such as SE33 [23].

### Influence of uracil DNA glycosylase on detection sensitivity

A slightly higher sensitivity in detecting degradation in samples treated with UNG compared to untreated samples was observed in Investigator Quantiplex^®^ Pro but not in PowerQuant^®^ System. This difference might be caused by the slightly higher difference in amplicon length between the autosomal and degradation targets in the Quantiplex^®^ Pro compared to the PowerQuant^®^ System with a 212 bp and 262 bp difference, respectively [14]. It is to be expected that higher amplicon differences between the two targets lead to stronger differences in PCR efficiency. Our findings confirm observations recently described by Holmes et al. [24], who observed generally higher DI values with Investigator Quantiplex^®^ Pro compared to PowerQuant^®^ System.

We explain the difference between samples treated with UNG and untreated samples in the Investigator Quantiplex^®^ Pro by the samples having suffered from deamination of cytosine due to hydrolytic damage, changing cytosine to uracil (e.g., [5]). UNG directly attacks uracil positions and eliminates uracil by cleaving the N-glycosidic bond between the base and the sugar-phosphate backbone of DNA, creating an abasic site in the DNA structure [25]. Such abasic sites comprise the weakest point in a DNA strand and enable the strand to break easily [9], for example, by heat stress during the first cycle of PCR. Changes in DI, however, were only minor. Further analyses of the correlation between degradation and storage time after UNG treatment, using a larger sample set and samples stored under different conditions, might provide interesting data on the detection of DNA degradation over time, which in turn might serve as a potential measure for the determination of the time since deposition of forensic traces.

### DNA degradation analysis by automated (pulse-field) electrophoresis

No significant correlation between DIN or GQN and storage time was observed in any of the conditions analyzed. Consequently, electrophoretic systems for assessing DNA integrity could not be found to provide a suitable alternative to quantitative real-time PCR and did not improve the detection of DNA degradation. Taking into account the ability of some highly sensitive qPCR kits, such as the PowerQuant^®^ System, to reliably point out samples that contain no or too little DNA for successful STR analysis [26], makes these kits highly useful in forensic genetics.

We did, however, observe a trend towards higher DIN numbers in samples stored at 37 °C compared to samples stored at RT and 4 °C. This trend is not statistically significant but correlates well with our previous findings of higher DNA recovery from samples stored at 37 °C compared to lower storage temperatures as described above.

## Key points

1. STR multiplex PCR analysis of forensic trace samples can show signs of DNA degradation even though the degradation index (DI) measured by quantitative real-time PCR is unobtrusive.
2. Degradation indices measured by quantitative real-time PCR showed no correlation with storage time in samples stored at three different temperatures for up to 316 days and no significant loss of DNA was observed.
3. Adding Uracil DNA glycosylase to enhance the sensitivity of detecting hydrolytic DNA damages improved the identification of DNA degradation. Thus, degradation effects other than fragmentation, such as deamination of DNA bases, play a role in reducing PCR efficiency of longer amplicons.
4. Electrophoretic methods did not improve degradation detection in forensic samples and are not superior over conventional quantitative real-time PCR.
5. Surprisingly, DNA recovery was significantly higher in samples stored at elevated temperatures (37 °C) compared to samples stored at room temperature or low temperatures (4 °C).
